# A qualitative exploration of decisions about dental recall intervals - Part 1: attitudes of NHS general dental practitioners to NICE guideline CG19 on the interval between oral health reviews

**DOI:** 10.1038/s41415-022-3998-z

**Published:** 2022-03-11

**Authors:** Hannah Scott, Anwen L. Cope, Fiona Wood, Natalie Joseph-Williams, Anup Karki, Emyr M. Roberts, Candida Lovell-Smith, Ivor G. Chestnutt

**Affiliations:** 4141594604001 Research Associate, Applied Clinical Research and Public Health, Cardiff University, University Dental Hospital, Heath Park, Cardiff, CF14 4XY, UK; 4141594604002 Honorary Lecturer in Dental Public Health, Applied Clinical Research and Public Health, Cardiff University, University Dental Hospital, Heath Park, Cardiff, CF14 4XY, UK; 4141594604003 Professor of Medical Sociology, Division of Population Medicine, School of Medicine, Cardiff University and PRIME Centre Wales, Heath Park, Cardiff, CF14 4YS, UK; 4141594604004 Senior Lecturer, Division of Population Medicine, School of Medicine, Cardiff University and PRIME Centre Wales, Heath Park, Cardiff, CF14 4YS, UK; 4141594604005 Consultant in Dental Public Health, Public Health Wales, Capital Quarter, Cardiff, CF10 4BZ, UK; 4141594604006 Principal Dentist, The Courtyard Dental Care, Cardiff, UK; Dental Practice Advisor, Cardiff and Vale University Health Board, Woodland House, Cardiff, CF14 4TT, UK; 4141594604007 Patient and Public Research Partner, Cardiff, UK; 4141594604008 Professor and Honorary Consultant, Dental Public Health, College of Biomedical and Life Sciences, Cardiff University and PRIME Centre Wales, UK; Clinical Director, University Dental Hospital, Cardiff and Vale University Health Board, UK; Director of Postgraduate Studies, School of Dentistry, Cardiff University, University Dental Hospital, Heath Park, Cardiff, CF14 4XY, UK

## Abstract

**Introduction** The National Institute for Health and Care Excellence (NICE) Guideline CG19 recommends that the intervals between oral health reviews should be tailored to patients' disease risk. However, evidence suggests that most patients still attend at six-monthly intervals.

**Aim** To explore facilitators and barriers to the implementation of CG19 in general dental practice.

**Methods** Semi-structured telephone interviews were conducted with 25 NHS general dental practitioners (GDPs) in Wales, UK. Transcripts were thematically analysed.

**Results** Dentists described integrating information on clinical risk, patients' social and dental history, and professional judgement when making decisions about recall interval. Although most GDPs reported routinely using risk-based recall intervals, a number of barriers exist to recall intervals at the extremes of the NICE recommendations. Many practitioners were unwilling to extend recall intervals to 24 months, even for the lowest-risk patients. Conversely, dentists described how it could be challenging to secure the agreement of high-risk patients to three-month recalls. In addition, time and workload pressures, the need to meet contractual obligations, pressure from contracting organisations and the fear of litigation also influenced the implementation of risk-based recalls.

**Conclusions** Although awareness of the NICE Guideline CG19 was high, there is a need to explore how risk-based recalls may be best supported through contractual mechanisms.

## Key points

- This study, which is reported in two parts, identifies barriers and facilitators to the implementation of NICE Guideline CG19 on intervals between oral health reviews.
- Describes current arrangements regarding dental recall interval settings and the opposition of dental practitioners to 24-month recall periods.
- Highlights that there are patient factors external to the control of dental practitioners that act as barriers to compliance with short recall intervals.

## Introduction

In 2004, the National Institute for Health and Care Excellence (NICE) published Clinical Guideline CG19 recommending that the interval between patients' oral health reviews should be determined according to their risk of dental disease. This includes consideration of the patient's medical, social and dietary history. NICE suggested that recall intervals should range from 3-24 months, depending on the outcome of the clinical assessment and that this should be discussed between dentist and patient.^1^

Despite this guideline having been published over 15 years ago, evidence suggests that many patients still attend at 6-9-monthly intervals.^2^ In 2012, a study reported that while 94% of responding dental practitioners were aware of the guideline, only 3% reported routinely recalling patients according to need.^3^ The authors concluded that factors such as dentists' lack of understanding of the guideline, concerns about late diagnosis (poor outcome expectancy), as well as patient resistance to change, may be contributing to poor adherence rates.

Policymakers in the UK have prioritised the implementation of risk-based recall intervals.^2^^,^^4^^,^^5^ It has been argued that the adoption of a risk-based approach to dental recall may foster the more efficient distribution of NHS dental resources, whereby patients are treated according to their dental needs and treatment requirements.^6^ However, a recent paper reported that almost half of all dental practices 'rely on regular check-ups for those in good oral health to achieve [contractual] targets'.^7^

This study, which is reported in two parts, sought to explore how decisions about dental recall intervals are made in general dental practice. Part one will describe the facilitators and barriers experienced by NHS general dental practitioners (GDPs) working in Wales towards the implementation of risk-based recall intervals. Part two will describe NHS patients' and GDPs' views regarding the potential role of shared decision-making (SDM) in a dental recall interval setting.

## Methods

A qualitative semi-structured telephone interview study involving NHS GDPs in Wales, UK was conducted between March and September 2019.

### Sampling and recruitment

GDPs were identified from a publicly-available database of NHS practices in Wales. The list was randomised and invitation letters sent until the required number of dentists had been recruited. In order to be eligible, dentists needed to spend at least 50% of their clinical time delivering NHS general dental care and not work at a practice yet involved in the Welsh NHS General Dental Service Reform Programme.

Based on Malterud *et al.*'s concept of information power,^8^ it was anticipated that approximately 20-30 GDPs would be recruited, in order to reflect a range of experiences and practicing environments. Data collection, transcription and analysis were undertaken concurrently to allow follow up of interesting topics. Following the completion of 25 interviews, two investigators judged that the richness of data within a theme no longer appeared to be increasing. Thus, no further interviews were conducted.

The study was reviewed and given a favourable ethical opinion by the Proportionate Review Sub-Committee of the East of England - Cambridge Central Research Ethics Committee (REC ref: 19/EE/0031). All participants provided written consent to participate in the study and to have their data used as part of the research and confirmed this verbally before interview.

### Data collection

Interview topic guides were prepared before data collection (Table 1; further details available on request). These were adapted over the course of the study as new findings emerged. Interviews were conducted by HS, a psychologist.

**Table 1:** Summary of the topic guide for GDP interviews

Topics	Prompts
Introductions and background	Aims of study; check outstanding questions; confirm consent
Context	Describing practice; describing characteristics of patient population
Risk-based dental recall intervals	Awareness of NICE guideline; familiarity; agreement. Extent of risk-based recall interval use; previous changes; anticipated changes; incentives to use risk-based recalls more. Advantages of risk-based recalls; disadvantages or concerns; impact on contract delivery
Perceived patient preference regarding recall intervals	Perceived patient preference; barriers to changing recall interval; facilitators to changing recall interval
Shared decision-making	Understanding of term; past experience; perceived importance; shared decision-making in different decisions; patients' desire to be involved in different decisions; advantages of shared decision-making; disadvantages of shared decision-making; barriers to shared decision-making; facilitators to shared decision-making; use of decision-making aids/resources
Shared decision-making regarding dental recall intervals	Patients' desire to be involved in recall interval decisions; information need to be involved

### Analysis

The interviews were transcribed verbatim and analysed according to the Braun and Clarke's principles of thematic analysis,^9^ supported by NVivo qualitative data analysis software (QSR International). Interviews were anonymised on transcription and checked against the original recording to ensure fidelity. A coding structure in which several 'codes' rested within a 'theme' was developed using a constant comparison method. In total, 20% of transcripts were double-coded by other members of the study team (FW and NJW) to test the fidelity of the coding structure. Any disagreements were resolved by discussion.

## Results

Just over half of participating dentists were men (14/25) and the majority (15/25) described themselves as dental associates (Table 2). The average time since qualification was 10.1 years (range 1-21 years). This was higher for practice owners (average 13.8 years) than associates (9.3 years). In total, 18 out of 25 dentists had qualified since the first publication of the NICE Guideline CG19 in 2004. Interviews generally lasted 20-30 minutes.

**Table 2:** Characteristics of participating GDPs

Characteristic	Frequency (n = 25)
**Sex**	
Female	11
Male	14
**Role in practice**	
Owner	8
Associate	15
Other	2
**Date of primary dental qualification**	
Pre-publication of NICE Guideline CG19 in 2004	7
Post-publication of NICE Guideline CG19 in 2004	18

The themes relating to attitudes to the NICE Guideline CG19, its implementation and attitudes towards risk-based recalls, are described below. Themes relating to the use of SDM in discussions about dental recall interval are presented in part two.

### NICE Guideline CG19 awareness and trust

All dentists were aware of NICE Guideline CG19. Although most were not familiar with the evidence that had informed their development, there was an implicit trust expressed in the NICE Guideline development process:

- *'I haven't seen the evidence for it, I've kind of taken it for its word really. To be honest, I haven't looked at the actual quality of the evidence'* (GDP4, male, dental associate, qualified post-CG19 publication)
- *'I think it's probably been well looked into and certainly in my group of patients with varied needs there are patients that, you know, they really don't need to come back every six months'* (GDP17, female, dental associate, qualified pre-CG19 publication).

### Agreement with CG19

While most dentists agreed that three-month recall intervals may be appropriate for patients at the highest risk of developing disease, most were uneasy extending the recall interval of low-risk, adult patients to the 24-month maximum recommended by the guideline. They believed that patients would be at risk of developing disease (particularly dental caries or oral cancer) and suffering adverse outcomes due to later diagnosis. As a result, many placed their lowest-risk patients on an annual or 18-month recall schedule:

- *'If I put somebody on a two-year recall and they came back and they had like, you know, their oral hygiene had deteriorated then they had loads of caries, I don't really feel like I was doing my job properly'* (GDP21, female, practice owner, qualified post-CG19 publication)
- *'Up to 12 months, no concern whatsoever. Above that, I'm always a little wary because of the risk of oral cancer […] it's the result of going on a course […] a lot of oral cancer now is appearing apparently in healthy individuals with no risk factors. And for the sake of fifteen minutes of their time once a year, on the National Health [NHS] it's about fourteen pounds thirty […] is it really worth that risk? I would say no'* (GDP23, male, practice owner, qualified post-CG19 publication).

There were a number of practitioners who expressed concern that NICE's decision to recommend risk-based recalls may be politically or financially motivated. A small number of other practitioners perceived that CG19, along with guidelines in general, reduced clinical autonomy and typically did not consider 'frontline' implementation issues:

- *'I kind of wonder, with NICE, whether it's all about sort of money, you know? Not actually interests of patients'* (GDP19, male, dental associate, qualified pre-CG19 publication)
- *'I kind of think that we've been pushed to standardise everything rather than, you know, judging on our own merits […] the people making the decisions for us to follow, they don't always work frontline so they don't really understand […] I feel like they're discounting our clinical judgement a little bit'* (GDP21, female, practice owner, qualified post-CG19 publication).

### Implementation of CG19

The majority of dentists reported that clinical risk assessment informed their recall interval recommendations for all patients and only a small number did not consider this to be part of their routine practice:

- *'We do literally use it every, every single day with every patient'* (GDP20, male, other, qualified post-CG19 publication).

Practitioners described how establishing patients' recall interval involved the synthesis of clinical findings, dental and social history, together with practitioners' clinical experience and personal knowledge of the patient. Dentists highlighted that recall intervals were not a 'tick box' exercise but required clinical insight and was typically more challenging in new dentist-patient relationships:

- *'I've been here 16 years, so I've got a very good idea. I could almost judge their check-up period before they walk in through the door […] I look at the caries, I look at the risk factors like dry mouth, smoking, age, how heavily restored they are. I look at these things and I'll make a number up really almost based on all that information. Assimilate it all and weighting it as I see appropriate […] I haven't got a system where it's like, one filling, you're coming back in twelve months, two fillings you're coming back in six'* (GDP16, male, practice owner, qualified pre-CG19 publication)
- *'We would do a risk assessment for their caries risk, their perio risk, their cancer risk and tooth surface loss risk. So you would base it on that, I think it's quite difficult as a foundation dentist sometimes to determine it because you haven't seen the patient long-term, it's only for a year. So you can't really judge that well how stable the patient is'* (GDP22, female, other, qualified post-CG19 publication).

### External barriers to implementation - patients' preferences

Dentists described tension between the implementation of CG19 and delivering care, which takes into account patient wishes about their recall interval. Most dentists believed that the majority of patients who regularly attended would prefer to come for check-ups more regularly than their risk profile may indicate. Many had experienced difficulty trying to change the recall of particular patients; usually when attempting to extend the recall of low-risk patients from 6-12-months. However, some acknowledged that reducing recall intervals to three months for high-risk patients also presented challenges and that these patients rarely attended at these intervals due to time, money and/or because irregular attendance patterns were more common among those with the highest rates of disease:

- *'"Is that okay, if I see you next year, will you be happy with that?" Most of them, they are [saying] "oh, you don't need to see me in six months?"'* (GDP3, female, dental associate, qualified pre-CG19 publication)
- *'If you try to put a patient on a three-month recall […] so they're paying every three months, they're going to be less keen. Some patients might not want the treatment, they're a bit anxious […] if they're going to go through a simple scale and polish or gum cleaning, they're going to want to go through it as less often as possible'* (GDP5, male, dental associate, qualified post-CG19 publication)
- *'Patients don't always want to take those three month recalls that they need […] we have a high rate of DNAs [do not attends] because quite often when that problem goes away, they just won't turn up'* (GDP1, female, practice owner, qualified post-CG19 publication).

### External barriers to implementation - contractual factors, workload pressures and litigation

Dentists described how contractual pressures and a target-driven culture within NHS general dental practice influenced their implementation of risk-based recall intervals. There was a recognition that regular six-month recalls for low-risk patients resulted in optimal generation of Units of Dental Activity (UDA). Some reported that placing higher-risk patients on three- or six-monthly recalls could also lead to additional Health Board-scrutiny. Several expressed concern that contract managers failed to consider the relative deprivation of the patient population when challenging practices about patterns of patient attendance and wished for greater respect for clinical autonomy:

- *'It makes it harder to deliver the NHS contracts. Because the nice patients with the lower risk, you want to see them regularly because they are easier to gain your UDA with, but you know, that's just, that's kind of cheating the system slightly isn't it?'* (GDP2, female, practice owner, qualified pre-CG19 publication)
- *'The NHS contract does play a part in how frequently you can ask a patient to come back as I feel that the Health Board are keeping an eye on that as well. So, if I have a patient, let's say, coming in every three months and they've been coming in every three months for the last two years, I really have to make sure in my notes it's documented exactly, exactly why I've kept them on a three-month recall instead of increasing them to a six-month or a nine-month'* (GDP25, male, dental associate, qualified post-CG19 publication).

Time constraints were also identified as a potential barrier to the assessment of individual risk within clinical encounters. Some dentists were concerned this would likely be exacerbated by ongoing NHS general dental service reform in Wales, while others were concerned about potential litigation arising as a result of extending recall intervals:

- *'To assess risk, you have to have had quite a bit of a conversation with a patient. The pressure of the practice is you don't have all the time in the world to do that. So you're relying on good assessment of risks with a small amount of information, which I suppose is risky in itself'* (GDP11, male, dental associate, qualified post-CG19 publication)
- *'I know I'm the generation that's been brought up with six-month check-ups, but we're also getting sued left, right and centre. If I say a year and I'm wrong, I'm in trouble'* (GDP13, male, practice owner, qualified pre-CG19 publication).

## Discussion

In light of increasing emphasis on the implementation of risk-based dental recall intervals within dental policy,^2^^,^^4^^,^^5^ this study sought to describe the attitudes of NHS GDPs working in Wales to the recommendations contained within the NICE Guideline CG19.^1^ It also aimed to describe factors which may facilitate or impede their use in practice. Within the present study, practitioners were familiar with the guideline and most reported routinely basing decisions about patients' recall interval on their risk of disease. Clinical judgement and consideration of patients' social and dental history was perceived to be important when making decisions about recall interval and practitioners felt aggrieved when this was challenged by organisations responsible for contractual monitoring. A number of barriers were identified to the implementation of recall intervals at the extremes of the NICE recommendations (3 and 24 months). The vast majority of practitioners were concerned that 24-month intervals posed an unacceptable risk to patients' oral health. Conversely, dentists described how it could be challenging to secure patient compliance with three-month recalls due to perceived patient preferences. This study provides some insight into why, 15 years after the introduction of the NICE Guideline CG19, the majority of NHS dental patients still attend at six-monthly intervals.^2^

Within medicine, a variety of factors have been described as influencing adherence to clinical guidelines. These include: awareness and familiarity; agreement; self-efficacy; outcome expectancy; ability to overcome the inertia of existing practice; and external barriers.^10^ In the current study, awareness of CG19 was high and although practitioners were often unsure of the evidence underlying the recommendations, they were familiar with their content. The fact that risk-based recalls had been part of the clinical guideline landscape for over a decade and a half appeared to diminish the role of inertia of existing practice. In general, practitioners felt able to use a risk-assessment process to determine an appropriate recall interval (self-efficacy) and felt confident extending the recall intervals of lower-risk patients from 6-12 months without deleterious effects on oral health (outcome expectancy). Conversely, factors which represented barriers to the implementation of risk-based recalls related to agreement with recall intervals at either end of the NICE-recommended timescale for adults (3 and 24 months), patient factors and features of the wider clinical environment, such as contractual monitoring, time and workload and clinical negligence liability.

The principal barrier to extension of recall intervals to 24 months for the lowest-risk patients were concerns about the development and/or progression of disease during this period. A recent Cochrane review reported that there was moderate-certainty evidence that there is likely to be little to no difference in terms of caries and gingival bleeding between 6-24-month recalls for adults over a four-year period.^11^ However, data to support this outcome came from one trial; while this trial was large and robustly conducted, it did not consider oral cancer development,^12^ which was cited by several dentists in the present study as a concern related to long recall intervals. Future research is therefore indicated to explore the effects of dental recalls at different intervals on the diagnosis of pre-malignant or malignant lesions and size and stage of oral cancers at presentation.

The use of qualitative interviews in this study facilitated the detailed exploration of GDPs' attitudes towards risk-based recall guidelines. Guided by accepted theories of sample size determination in qualitative enquiry,^9^ investigators judged that after 25 interviews further data collection was unlikely to yield substantial new findings. Nevertheless, it is not possible to state with certainty that the views of the participants of this study reflect the full spectrum of attitudes among NHS GDPs in Wales, or that these findings can be confidently generalised to other settings. It is not known whether the recall intervals of GDPs who participated in the study are indicative of the wider profession. It is plausible, however, that the barriers and facilitators to the implementation of risk-based dental recall guidelines reported in this study could contribute to improving the understanding of why so many patients still attend for oral health reviews at close to six-monthly intervals.

If policymakers and bodies responsible for the organisation of dental services continue to place emphasis on the implementation of risk-based recall intervals and use these within contract monitoring arrangements, it should be with an awareness that there are patient factors external to the control of dental practitioners that act as barriers to compliance with short recall intervals. It has been recognised that there are financial disincentives within current NHS general dental service contracting arrangements against increasing the recall intervals of low-risk patients.^7^ Therefore, to fully realise the potential of more equitable and effective distribution of NHS dental resources, commissioners will need to recognise and accommodate barriers to the use of the full range of recommended recall intervals.

## Conclusions

A decade and a half after the original publication of CG19, it appears from this study that risk-based dental recall intervals may become a routine clinical practice for the majority of practitioners. However, there is widespread lack of agreement with 24-month recall intervals and a recognition that NHS contracting arrangements may perversely incentivise retaining six-month recalls for low-risk patients. Conversely, barriers to three-month recall intervals for the highest-risk patients include patient preferences and contractual monitoring arrangements. More work is needed to explore the effects of longer recall intervals on clinical outcomes associated with oral cancer and to describe how risk-based recalls may be best supported through contractual mechanisms.
